# High energy electron diffraction instrument with tunable camera length

**DOI:** 10.1063/4.0000240

**Published:** 2024-03-25

**Authors:** P. Denham, Y. Yang, V. Guo, A. Fisher, X. Shen, T. Xu, R. J. England, R. K. Li, P. Musumeci

**Affiliations:** 1 Department of Physics and Astronomy, UCLA, Los Angeles, California 90095, USA; 2 Department of Engineering Physics, Tsinghua University, Beijing 100084, China; 3 SLAC National Accelerator Laboratory, Menlo Park, California 94025, USA

## Abstract

Ultrafast electron diffraction (UED) stands as a powerful technique for real-time observation of structural dynamics at the atomic level. In recent years, the use of MeV electrons from radio frequency guns has been widely adopted to take advantage of the relativistic suppression of the space charge effects that otherwise limit the temporal resolution of the technique. Nevertheless, there is not a clear choice for the optimal energy for a UED instrument. Scaling to beam energies higher than a few MeV does pose significant technical challenges, mainly related to the inherent increase in diffraction camera length associated with the smaller Bragg angles. In this study, we report a solution by using a compact post-sample magnetic optical system to magnify the diffraction pattern from a crystal Au sample illuminated by an 8.2 MeV electron beam. Our method employs, as one of the lenses of the optical system, a triplet of compact, high field gradients (>500 T/m), small-gap (3.5 mm) Halbach permanent magnet quadrupoles. Shifting the relative position of the quadrupoles, we demonstrate tuning the magnification by more than a factor of two, a 6× improvement in camera length, and reciprocal space resolution better than 0.1 Å^−1^ in agreement with beam transport simulations.

## I. INTRODUCTION

Ultrafast electron diffraction (UED) enables following the dynamical evolution of matter at fundamental atomic spatial and temporal scales. The method involves exciting a sample (typically using an ultrafast laser pulse) and probing its structure at later times with a very short electron bunch to capture a snapshot of the sample reciprocal space in a diffraction pattern on a far-field screen.^1–3^ The potential of ultra-fast electron scattering techniques in exploring atomic-scale structural dynamics has been demonstrated in several experiments ranging from ultrafast phase transitions, warm-dense matter, correlated electron systems, diffuse scattering, gas-phase, and liquid phase studies.^4–7^

While the technique's early implementations have been at non-relativistic electron energies,^8–10^ it was soon recognized that carrying out the experiments with relativistic electrons would bring significant advantages especially in coping with the effects of space charge which would limit either the number of electrons or the temporal duration of a single bunch.^11–13^ Further introduction of methods from accelerator physics in recent years such as radio frequency (RF) compression,^14^ advanced cathodes,^15^ phase space manipulations,^16,17^ and better detectors^18,19^ have all contributed to the significant improvements in the performance of modern UED instruments.

An area of focus for advancement involves the evolution of magnetic optics for UED. Upstream of the sample, strong lenses can be used to focus a high brightness electron beam to sub-micron spot sizes to investigate the dynamics of very small or heterogeneous samples.^20,21^ After the sample, preliminary experiments have shown that the use of a magnetic lens can improve the reciprocal space resolution.^22,23^ However, most beamlines still utilize a propagation drift to convert the scattering angles into a spatial offset at the detector.

Post-sample magnetic optics would also allow to greatly extend the energy of UED instrumentation. Relativistic UED beamlines have been limited to kinetic energies <4 MeV for many practical reasons such as the available RF power and accelerating gradient in the RF gun. Another important limitation comes from the fact that due to the reduction in scattering angles, higher energies necessitate longer drifts to separate the scattered electrons from the main beam on the detector screen. At the same time, it has been pointed out that the diffraction contrast from the relative coherence length (i.e., the ratio between the electron wavelength and the beam intrinsic divergence) does not suffer from increasing the beam energy.^3^ This occurs since geometric emittance and De Broglie wavelength scale inversely with the normalized beam longitudinal momentum βγ. A higher beam energy in UED carries several other advantages. The flatter Ewald sphere resulting from the shorter wavelength of the scattering wave brings strong sensitivity to very high orders in the diffraction pattern. For gas phase, the contribution of velocity mismatch is further suppressed as the deviation from the speed of light scales as $\gamma^{-2}$. Most importantly, higher beam energies are associated with higher penetration depth. The capability to go through thicker samples could significantly enhance the range of experiments accessible to UED, paving the way to liquid cells, shock dynamics and diffuse scattering studies. Finally, at the cost of increased complexity, beamlines at higher electron energy can pack more electrons in tighter spots and shorter bunch lengths due to the substantial *γ* dependence in space charge effects.

In this paper, we investigate, with the help of simulations and in a proof-of-principle experiment carried out at the UCLA Pegasus beamline,^24^ the addition of post-sample optics to push the energy in UED instrumentation to 8 MeV (nearly twice the previous state-of-the-art^3^). To do this, we implement a two-lens telescope where one of the optics is a strong permanent magnet-based quadrupole (PMQ) triplet to restore the diffraction contrast and maximize the angular magnification on a screen located 1 m from the sample. Controlling the relative position of the PMQs allows for the demonstration of tunable diffraction camera length and improvement of the reciprocal space resolution. In principle, round solenoidal lenses could be used for this task. Still, short focal length at >5 MeV electron energies can only be achieved using extraordinarily high magnetic fields and bulky and/or superconducting coils.^25^ By replacing solenoids with quadrupole triplets, we separate the horizontal and vertical optical degrees of freedom and add a layer of complexity in optimization and alignment but greatly simplify the system in terms of cost and size.

We will start the discussion by reviewing the quadrupole optimization strategy informed by a preliminary thin lens system description. Details are then provided on experimental techniques for aligning the PMQ triplet to a level sufficient to achieve the required optical focusing with minimal steering effects on the beam. Finally, experimental results using the high brightness beam from the Pegasus beamline demonstrate tunable magnification of the diffraction pattern from a crystalline gold sample at 8.2 MeV in a 1 m distance. Interestingly, we can assess the imaging performance of the quadrupole triplets employed in the experiment by studying the diffraction pattern distortions as the alignment through the optical system is varied. Once evaluated, the aberrations can be cross-referenced with 3D magnetostatic simulations, enabling the identification of the underlying factors contributing to the quality of the lenses.

## II. THIN LENS OPTICS DESCRIPTION AND NUMERICAL OPTIMIZATION OF QUADRUPOLE OPTICS

### A. Thin lens description of angular magnification

**FIG. 1.**
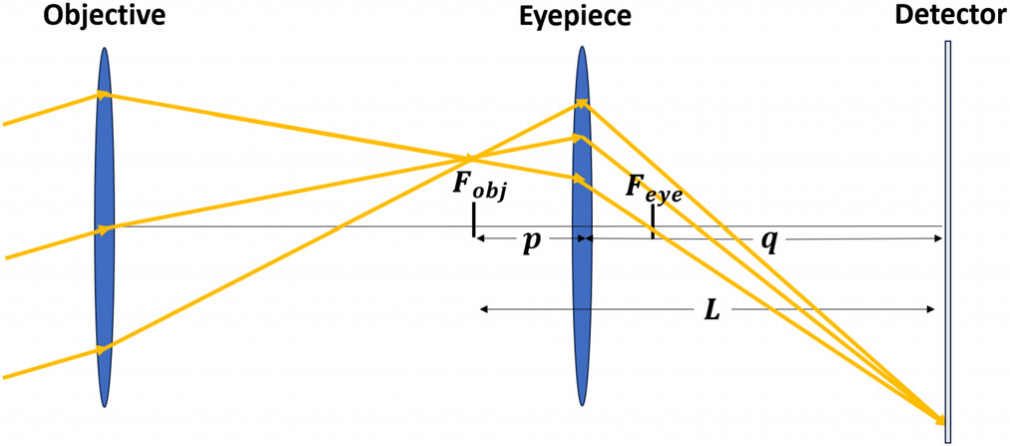
Ray diagram showing an angular magnification telescope. Initially, parallel rays converge to the same position at the detector with a magnified offset compared to the objective's back focal plane.

The magnification of the optical system after the UED sample can be easily understood using thin lens transport matrices. Consider two stages; the first is an objective lens, with focal length *f_o_*, followed by a drift distance equal to its focal length, which transports to the back focal plane. The second is an eyepiece lens, with focal length *f_e_*, which images the back focal plane of the objective lens to a downstream detector plane with magnification *m*. The matrix transport relating initial coordinates $\left(x_{0},x_{0}^{\prime }\right)$ to the final coordinates $\left(x_{f},x_{f}^{\prime }\right)$ is given by

$$\left(\begin{array}{c} x_{f}\\ x_{f}^{\prime } \end{array}\right)=\left(\begin{array}{cc} m & 0\\ m^{\prime } & \frac{1}{m} \end{array}\right)\left(\begin{array}{cc} 1 & f_{o}\\ 0 & 1 \end{array}\right)\left(\begin{array}{cc} 1 & 0\\ -\frac{1}{f_{o}} & 1 \end{array}\right)\left(\begin{array}{c} x_{0}\\ x_{0}^{\prime } \end{array}\right),$$ (1)

where $m^{\prime }$ is the axial derivative of *m* at the image plane.^26^ A ray diagram of this transport is shown in Fig. 1.

If the eyepiece lens is imaging over a total distance *L*, the object and image distances *p* and *q* can be expressed in terms of *L* and *f_e_* as $\frac{L}{2}\left(1\pm \sqrt{1-4f_{e}/L}\right)$, respectively. For a strong eyepiece lens (i.e., $f_{e}\ll L$), we can approximate the magnification $m\approx -L/f_{e}$.

In this case, the relationship between the final position on the detector screen and the initial diffraction angle can be written as

$$x_{f}=mf_{o}x_{0}^{\prime }\approx -L\left(f_{o}/f_{e}\right)x_{0}^{\prime },$$ (2)

clearly showing how a tunable diffraction camera can be obtained by adjusting the focal lengths for the eyepiece and objective in the angular magnification telescope.

### B. Quadrupole optics

In implementing the scheme, we use two quadrupole triplets as objective and eyepiece, respectively. For the eyepiece, where it is essential to use as strong of a lens as possible to maximize the magnification in a given distance, we take advantage of high gradient permanent magnet quadrupole (PMQ) lenses, which have been shown to have a focal length of few cms at MeV electron energy.^27^ An additional benefit of using quadrupole-based lenses, which results from the independent evolution of the rays in the horizontal and vertical planes, is that the scheme offers a wide range of imaging solutions with different magnifications, all accessible with relatively small translations of the elements within the eyepiece triplet, which is critical given the lack of tunability of the PMQ gradients.

The optimization of the strengths of the quadrupoles in the objective lens and the eyepiece PMQ positions is based on a matrix representation of the transport from the sample to the detector. The focusing matrices include an approximation for the quadrupole fringe fields where an Enge function fits the gradient for each quad as

$$G(z)=\frac{G_{0}}{2}\left[\text{tanh}\left(\frac{b}{2}\left(\frac{l}{2}-z\right)\right)+\text{tanh}\left(\frac{b}{2}\left(\frac{l}{2}+z\right)\right)\right],$$ (3)

where *l* is the effective length of the quadrupole, *b* is the steepness parameter of the edges, approximately half the quadrupole gap radius, and *G*_0_ is the nominal peak magnetic field gradient.^28^

In the paraxial approximation, the linearized transverse equations of motion are given by

$$x^{\prime \prime }+\kappa (z)x=0,$$ (4)

$$y^{\prime \prime }-\kappa (z)y=0,$$ (5)

where primes are derivatives with respect to the propagation distance *z*, the focusing strength $\kappa (z)=\frac{G(z)}{\left[B\rho \right]}$, and [Bρ]=p/e is axial momentum normalized by the electron charge.^29^ In contrast, the focusing strength for a round solenoid is inversely proportional to the square of [Bρ].

The general solution to the ray equation is of the form

$$x(z)=x_{0}C_{x}(z)+x_{0}^{\prime }S_{x}(z),$$ (6)

$$y(z)=x_{0}C_{y}(z)+x_{0}^{\prime }S_{y}(z),$$ (7)

where the functions $C_{x},C_{y},S_{x}$, and *S_y_* are the transport cosine and sine-like trajectories (also known as principal rays), the principal planes of the quadrupole optics are defined by where these functions are zero; for example, a focal plane is where *C* = 0 and an imaging plane occurs whenever *S* = 0 (where a subscript of x or y is implied). The ideal optical functions for diffraction are *C* = 0 and *S* as large as possible to maximize the reciprocal space resolution.

### C. The role of post-sample optics in improving diffraction resolution

To estimate the effect of the magnification on the reciprocal space resolution of a diffraction pattern, it is useful to define a diffraction contrast ratio, R=σ/q, which is the RMS size of the beam, *σ*, at the detector, divided by *q*, which is the distance separating a Bragg peak from the direct beam. The Bragg separation *q* is related to the Bragg angle, denoted by *θ_B_*, and angular magnification, denoted as *S*, through the equation $q=S\theta_{B}$. Since *θ_B_* depends on the particular reciprocal space plane we are looking at, the diffraction contrast ratio can be normalized by the lattice periodicity to obtain the momentum transfer or q-resolution

$$\sigma_{Q}=\frac{\sigma }{S\lambda },$$ (8)

where *λ* is the De Broglie wavelength of the electrons illuminating the sample.

Assuming the transverse phase space of the beam at the sample plane is uncorrelated (in general, the correlation is very small for nearly parallel beam illumination, and adding it does not change the conclusion of this section), the width of a Bragg peak, as measured on the detector, is related to the initial conditions of the beam at the sample plane by

$$\sigma =\sqrt{C^{2}\sigma_{0}^{2}+S^{2}\sigma_{\theta }^{2}+PSF^{2}},$$ (9)

where *σ*_0_ and $\sigma_{\theta }$ are the initial RMS spot size and angular divergence of the beam at the sample, respectively, and *PSF* is the point spread function of the screen.

Inserting this expression into the relative q-resolution, we obtain

$$\sigma_{Q}=\frac{\sigma_{\theta }}{\lambda }\sqrt{1+{\left(\frac{C\sigma_{0}}{S\sigma_{\theta }}\right)}^{2}+{\left(\frac{\mathrm{PSF}}{S\sigma_{\theta }}\right)}^{2}}.$$ (10)

When there are no post-sample optics, *S* is just the drift length and *C* = 1 and the best choice to improve the resolution is to increase the drift length from the sample to the detector to minimize the two contributions in the quadrature sum that multiply $\sigma_{\theta }/\lambda$. Note that for high energy beams the intrinsic beam divergence scales as $\beta \gamma^{-1}$, and longer distances are needed to approach this ideal resolution.

**FIG. 2.**
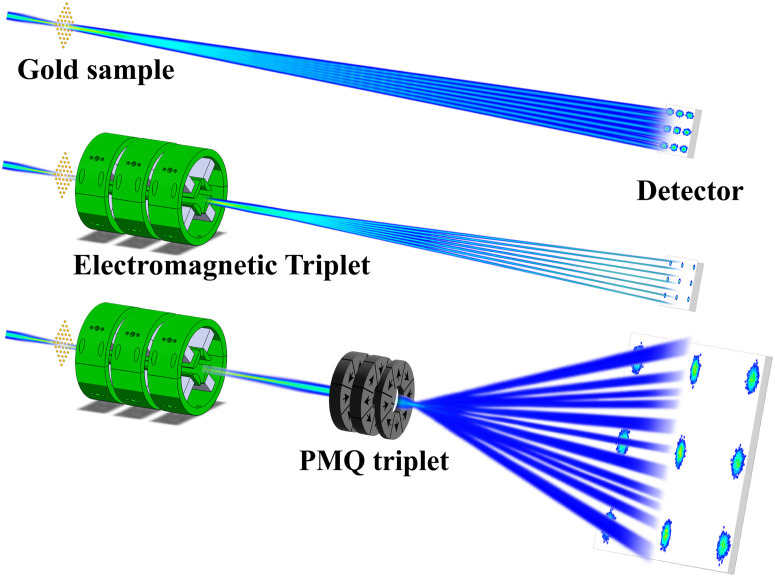
Cartoon depicting the angular magnification telescope concept implemented using two quadrupole triplets as applied to the Pegasus UED beamline.

The benefit of using post-sample optics is that we can arrange it to have *C* = 0 at the detector plane and then, for higher beam energies, greatly increase the angular magnification *S* to minimize the contribution from the *PSF* as illustrated in Fig. 2. The figure shows how adding an objective triplet (b) sharpens the diffraction pattern (essentially by zeroing *C*), and adding the PMQ eyepiece (c) magnifies the pattern by increasing *S* and spreading out the Bragg peaks.

### D. Momentum mapping conditions

The matrix representation for the linear optical transport was first used to numerically optimize the quadrupole gradients/currents and PMQ spacings to obtain equal magnifications at the detector plane. The parameters from the linear optimization were then used in start-to-end GPT simulations, which utilized actual 3D field maps from the quadrupoles from magnetostatic simulations. The results were found in excellent agreement with the idealized fringe field matrix model.

**FIG. 3.**
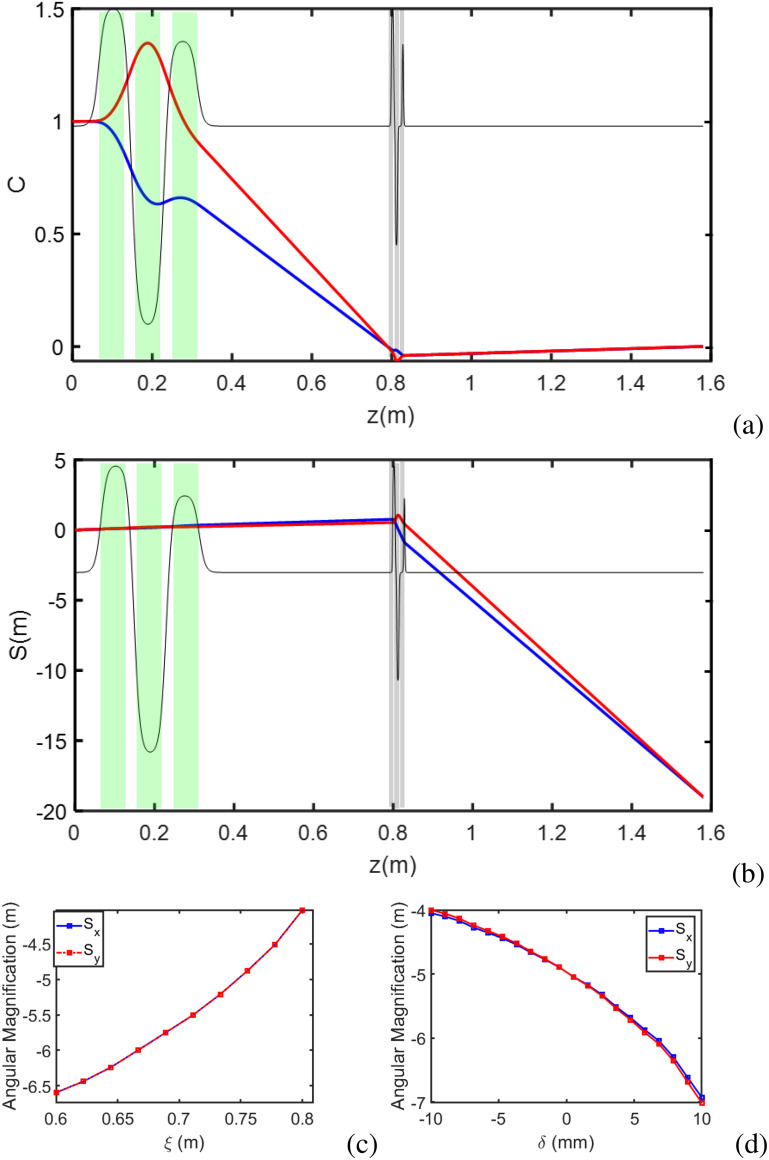
(a) The cosine-like principal ray is depicted traversing an optimized optics setup where the green quadrupole triplet positions back focal planes just in front of the PMQ triplet. The PMQ triplet then forms images on the downstream detector at z=1.6 m. (b) The sine-like principal ray is magnified at the detector. (c) An optimization scan for angular magnification is conducted for a nearby screen, with symmetric back focal planes set at varied positions while the PMQ triplet maintains imaging at z=1.02 m. (d) Back focal planes are separated using the green quadrupole triplet, and the spacings of the PMQ triplet are optimized to restore symmetric imaging at z=1.02 m.

In Figs. 3(a) and 3(b), we show such an optimized set of solutions for the cosine-like and sine-like trajectories, plotting the principal rays obtained from the matrix transport. In Fig. 3(a), note the axis is unitless in accordance with Eqs. (6) and (7). In this optimization, the positions of the back focal planes are placed symmetrically just in front of the eyepiece, which is 0.8 m downstream of the sample position (which defines *z* = 0 in this plot) while the detector screen is at *z* = 1.6 m. In the optimization, the currents in the objective triplet are limited to avoid overheating and magnetic field saturation in the yoke. Similarly, we restrict the range of possible spacings of the PMQs since they are mounted on a flexure-based stage that can provide only mm-range adjustability.

At the final screen, the system can achieve *S* = −20 m, more than one order of magnitude larger than what would be achieved without post-sample optics. In fact, at this plane, the magnification would be so large that a very large detector screen would be needed to capture most of the diffraction peaks (i.e., a Bragg angle of 0.5 mrad would translate in an offset of 1 cm). For this reason, the experiment was conducted at the nearby screen located at *z* = 1.02 m from the sample where lower magnification factors could be obtained so that multiple Bragg peaks could fit on the detector screen.

There are two ways to tune the magnification for a fixed distance from the sample to the detector. In one case, we can vary the quad currents so that both back focal planes of the green triplet objective lens occur at the same longitudinal position scanned longitudinally along with the PMQ triplet. The results at the nearby screen are shown in Fig. 3(c) where *ξ* is the position of the objective back focal planes. For each solution, the eyepiece is optimized to image onto the detector, yielding a tunable overall angular magnification at the *z* = 1.02 m mark. This approach requires the PMQ stage to move over a long range, which is not feasible to install in the beamline vacuum.

Alternatively, we can leave the PMQ triplet central position fixed and utilize the variability of spacings between PMQs to image asymmetric objective back focal planes with equal magnifications onto the detector. Results of this strategy are shown in Fig. 3(d). The parameter *δ* represents the separation of the two objective lens back focal planes about a fixed *ξ* = 0.75 m position; then, the eyepiece spacings are solved to restore symmetric angular magnification.

## III. PMQ TRIPLET SETUP AND ITS ALIGNMENT

**FIG. 4.**
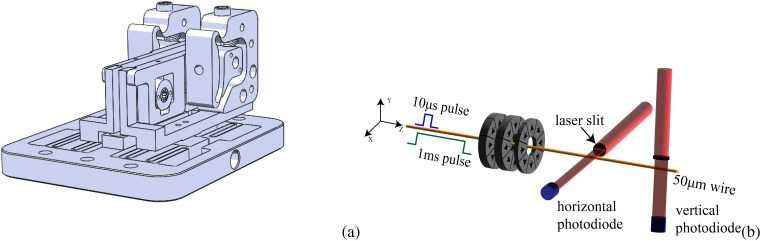
(a) Technical drawing of the PMQs as mounted on the flexure stage. (b) Setup of the pulsed-wire alignment technique.

Each PMQ is a 16-sector Halbach-style array featuring an inner diameter of 3.5 mm and an outer diameter of 7 mm.^27^ They are formed using wire electrical discharge machined N35SH NbFeB. The PMQ triplet is positioned on a flexure-based mounting stage, which keeps the central quad fixed in position with respect to the stage, but allows for the capability to fine-tune both upstream and downstream PMQ spacings [refer to Fig. 4(a)]. Theoretically, this design allows us to achieve optimal imaging conditions by precisely adjusting the longitudinal distances between the quadrupoles.

The peak gradients and effective lengths of the assembly were initially derived from *Radia*^30^ simulations and subsequently reevaluated using Hall probe measurements on the manufactured PMQs. The PMQs' gradients consistently align with values documented in earlier research which originally used the same triplet as this study.^27^ The most recent measurements of PMQs' magnet parameters are reported in Table I.

**TABLE I.** PMQ parameters.

	Gradient (T/m)	Eff. length (mm)
First quadrupole	510	6.16
Second quadrupole	518	6.16
Third quadrupole	417	3.9

Achieving the specified imaging condition required precise pre-alignment of the PMQ triplet on the stage. In the setup, the mounts for the upstream and downstream PMQs have horizontal and vertical fine adjustment micrometers. A pulsed-wire method^31,32^ was employed to ensure the relative alignment of the elements in the triplet.

The technique is illustrated in Fig. 4(b) and works as follows. A beryllium-enhanced copper wire, measuring 50 *μ*m in diameter, is threaded through the aperture of the PMQ triplet and carefully tightened. Subsequently, a pulse generator transmits a square-wave electric signal through the wire. Owing to the magnetic fields of the PMQs, the wire experiences a kick, the strength of which is proportional to its displacement from the physical center of the PMQs. The kick travels through the wire and is detected by a laser-photodiode system which translates the wire displacement into a voltage signal that can be recorded on a scope.

**FIG. 5.**
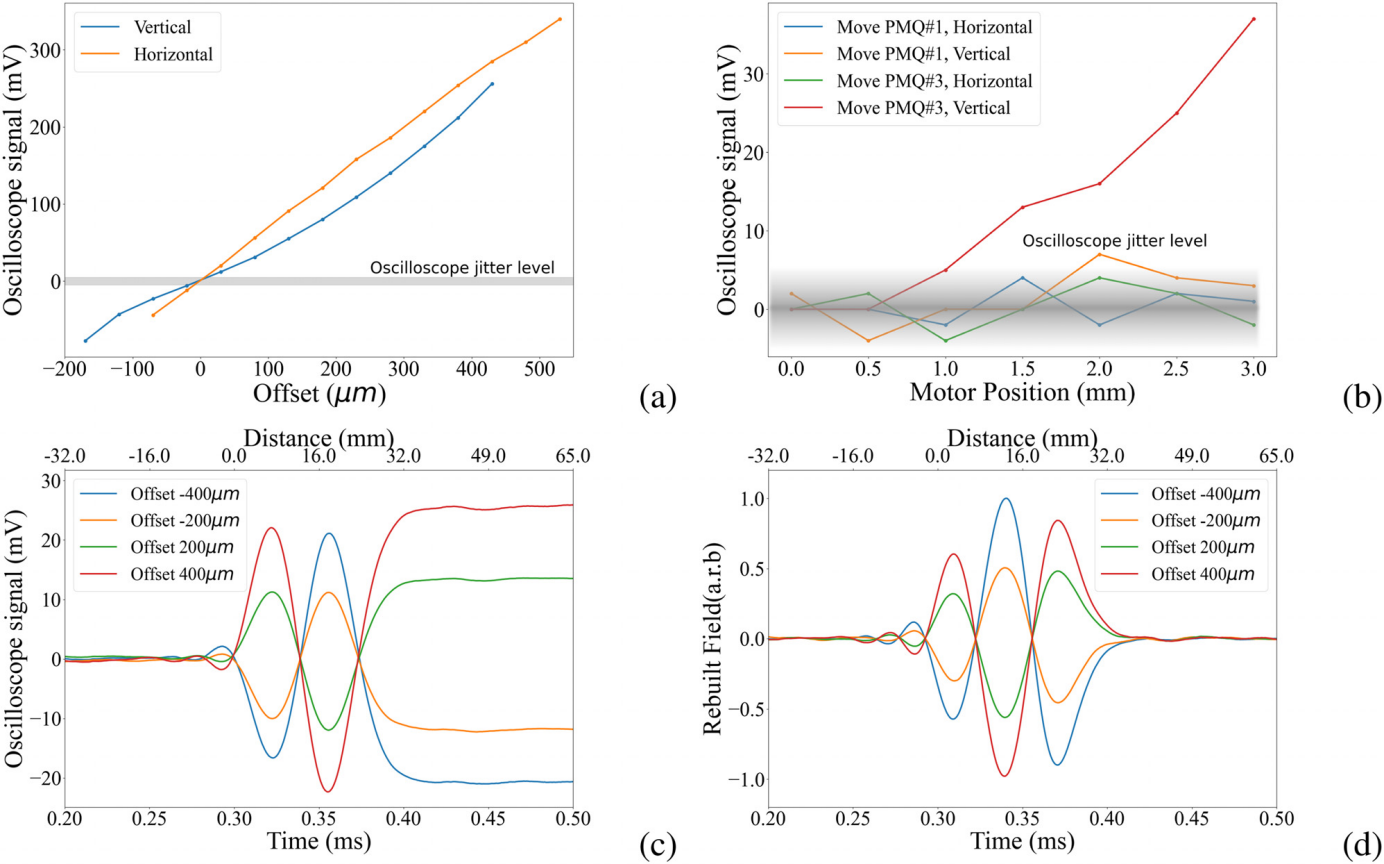
(a) Calibration of oscilloscope signal of central peak with the PMQs' displacement. (b) Oscilloscope signal of the central peak when the first and third PMQs are moved. (c) Signal with different transverse offset. The bottom axis is the signal timing, and the top axis is the corresponding distance. (d) Retrieved PMQ field from pulsed-wire signal.

When a long 1 ms current pulse is employed, the photodiode trace conveniently represents the second integral of the field, i.e., the trajectory of the electrons. Varying the position of the PMQ holders yields a linear relationship between the peak signal height of the kick and the PMQ position (as expected, the field grows linearly when going off-axis). Therefore, the PMQs can be aligned by flattening the signal trace. The accuracy level of the pulsed-wire method is determined by the photodiode conversion gain, string tension, power supply jitter, and ultimately by the oscilloscope readout error. In Fig. 5(a), we can see a linear correlation between signal height and actual offset of PMQs. Given the oscilloscope readout jitter of 5 mV after averaging over 128 data points, we estimate the alignment accuracy obtained with this method to be less than 25 *μ*m. Following static alignment, we conduct tests to verify that the alignment is preserved when axially translating the upstream and downstream PMQs. Our findings in Fig. 5(b) indicate that the PMQs remain generally aligned, although a slight horizontal offset is observed for the last PMQ. The maximum signal we collected is 34 mV, which, applying the calibration from Fig. 5(a), indicates that the misalignment of the PMQs is limited to 50 *μ*m.

When utilizing shorter pulses, the signals arise from the effective velocity kick in the PMQ fields (i.e., first integral). The output signal for different transverse offset is shown in Fig. 5(c). The traces can be numerically derived to retrieve the actual field profiles for different wire positions. Figure 5(d) shows the result of the fields retrieved from the photodiode traces using a 10*μ*s pulse. As expected, the peak field changes sign and increases in magnitude depending on the offset of the wire from the alignment axis. Upon further inspection, it is found that the peak fields change linearly with transverse offset in the quadrupoles.

## IV. EXPERIMENTAL RUN

**FIG. 6.**
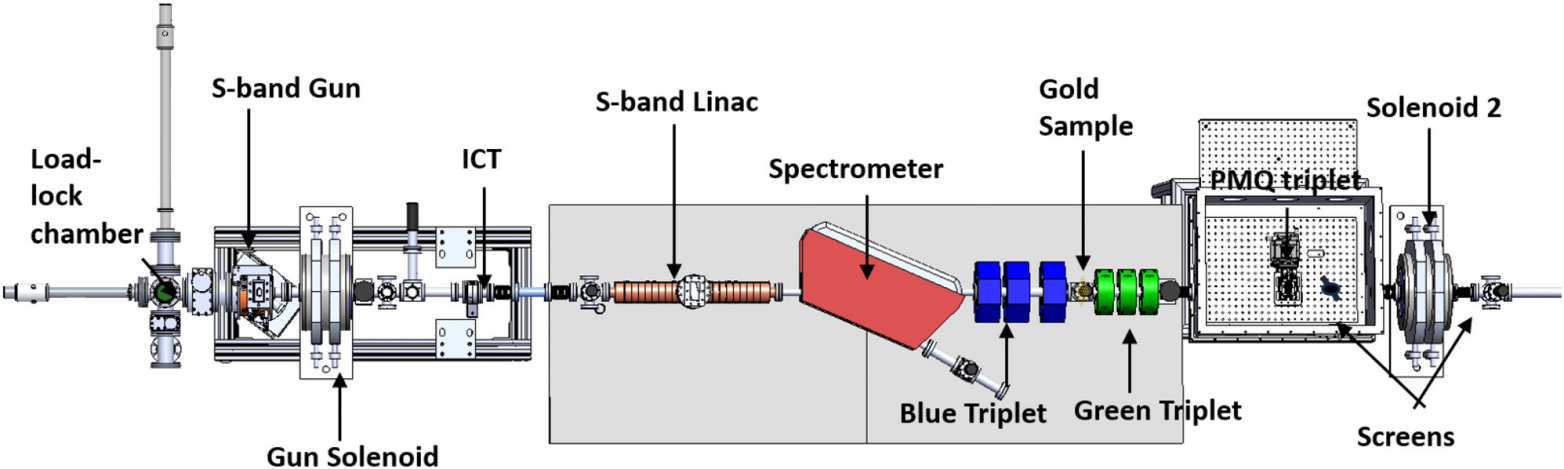
Pegasus beamline technical drawings showing the main elements in the experimental setup.

The experiment was performed at the UCLA Pegasus Laboratory, which is a high brightness beamline based on a high gradient 1.6 cell S-band RF photo-injector gun.^33^ A top-down view of the PEGASUS experimental setup is shown to scale in Fig. 6. The photo-injector is operated with low charge of 0.5 pC per bunch as measured by an integrated current transformer (ICT) located at the gun exit. The laser spot was focused at normal incidence on an alkali antimonide photocathode by a 0.75 m focal length lens to a size of 150 *μ*m. The gun rapidly accelerates the beam to 3.2 MeV, effectively mitigating transverse phase space degradation caused by space charge. The beam can be accelerated by a dual slot resonantly coupled high shunt impedance linac to 8.2 MeV kinetic energy. A waveguide switch can be used to cut off the RF power to the linac. The gun solenoid in combination with the blue quadrupole triplet are used to minimize the RMS angular spread at the sample plane. A 30 nm thick single crystal gold foil diffraction standard mounted on a 3 mm TEM grid holder was placed in the beamline 3.1 m downstream of the cathode as a sample. A HeNe laser copropagating with the electron beam was used to align the sample and the axes of the objective and eyepiece lenses. Referring to Fig. 3, the green triplet center is located 0.19 m from the sample and the PMQ triplet that serves as the eyepiece to magnify the back focal plane of the objective lens is located 0.81 m from the sample plane.

**FIG. 7.**
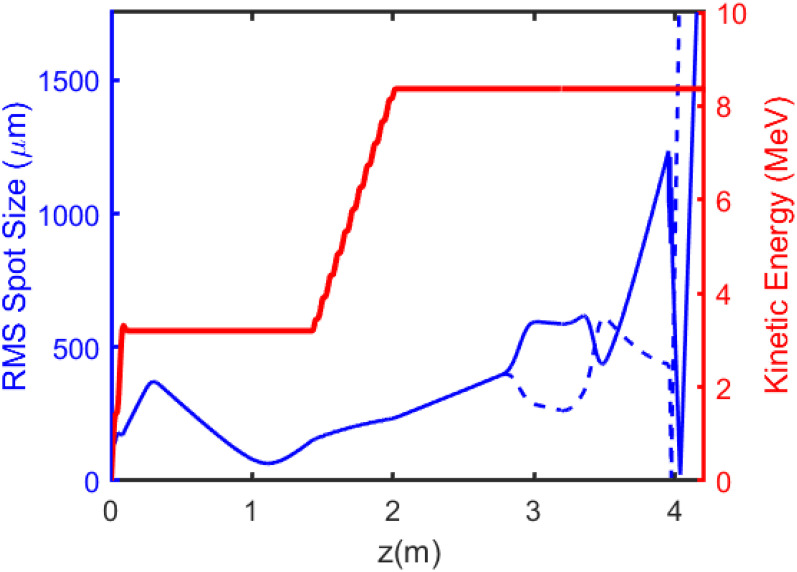
Results from self-consistent start-to-end GPT simulation of the Pegasus beamline including RF gun, linac and space charge effects. The rms horizontal (blue solid) and vertical (dashed) envelopes and the kinetic energy (red) are shown up to the detector.

Start-to-end simulations were conducted for the beamline setup using the particle tracking software GPT.^34^ GPT considers space charge effects and can use actual field maps for the beamline elements thus incorporating higher-order aberrations. The simulations also factor in the beam acceptance through the PMQ triplet aperture. Diffraction at the sample is modeled by applying diffractive kicks to the simulated phase space beam distribution at the sample plane. Figure 7 illustrates the evolution of the RMS transverse sizes of the beam and kinetic energy up to the last detector plane.

**FIG. 8.**
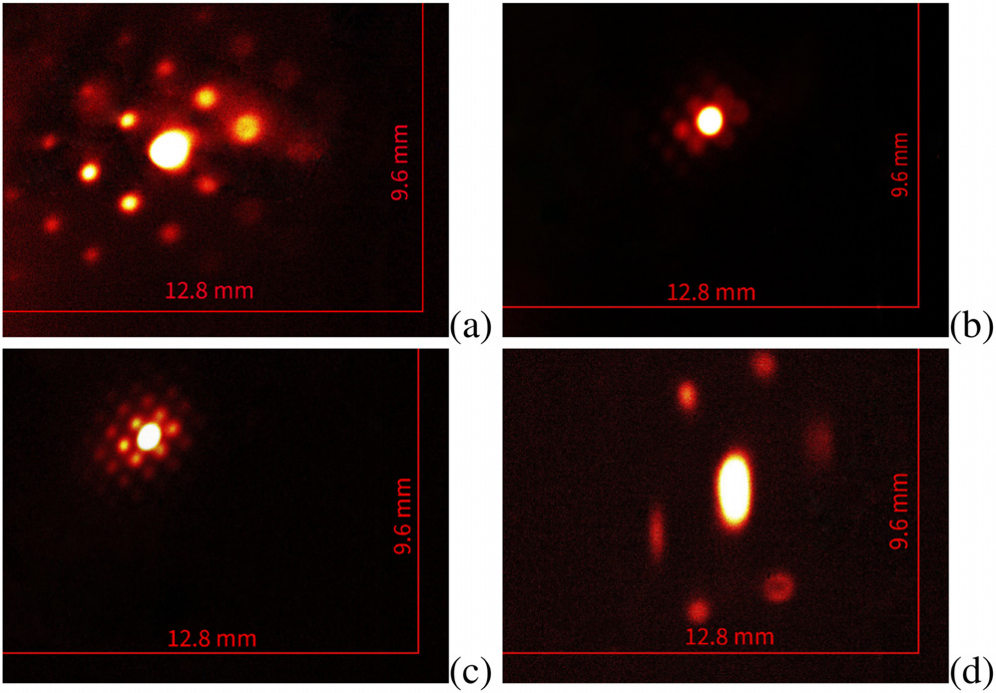
Single-shot diffraction patterns captured at the *z* = 1.02 m fluorescent screen. For (a) the linac is turned off and the kinetic energy is 3.1 MeV. The green quadrupole triplet is tuned to focus on the detector. (b)–(d) are acquired with the linac turned on and the beam energy at 8.2 MeV, (b) is obtained without focusing, (c) with focusing, and (d) with magnification.

During the experimental run, we implemented the same optimization strategy as in the simulation. Initial diffraction images were captured for reference with the linac switched off at a screen inside the vacuum box, while the beam energy was measured at 3.1 MeV and a representative pattern is shown in Fig. 8(a). Subsequently, the linac was activated and operated at an accelerating phase, raising the beam energy to 8.2 MeV while minimizing energy spread. In Fig. 8(b), we show the diffraction pattern recorded in this configuration without optics after the sample. Moving to Fig. 8(c), the green quadrupole triplet was first optimized to position the back focal plane at the first DRZ screen located 22 cm downstream of the PMQ triplet. By monitoring this screen, we further optimized the green triplet to bring the back-focal planes in front of the eyepiece and then inserted the PMQ triplet onto the beam axis. The relative spacings between the PMQs were then optimized to symmetrize the final image, resulting in a strongly magnified diffraction pattern shown in Fig. 8(d). It is immediate to note the large magnification imparted by the eyepiece. It is also clear that only 6 Bragg peaks were propagated through the small PMQ aperture, in agreement with the prediction of the simulation once the actual PMQ stay-clear size was included in the model. In a cylindrically symmetric system, we would expect to observe on the detector screen the lowest order 8 Bragg peaks of the cubic gold lattice, but the astigmatic transport in x and y resulting from the use of quadrupole lenses, and the particular rotation of the sample, causes two of the four 200 Bragg peaks to fail to clear the tight aperture of the PMQ magnet, preventing their transport to the detector screen. By employing a steering magnet at the entrance of the vacuum box, we could direct different portions of the diffraction pattern through the PMQ triplet and display different sections of the reciprocal space.

**FIG. 9.**
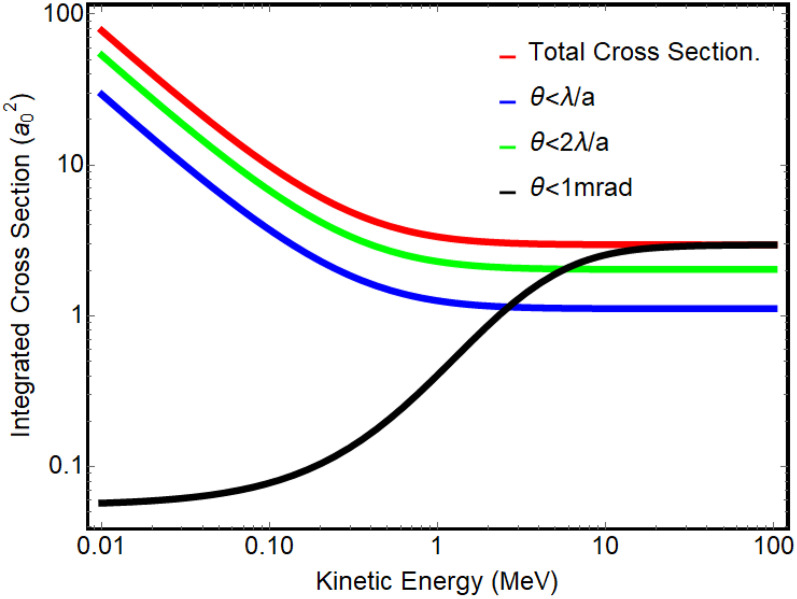
A comparison of integrated cross sections for Au in the range 0.01–100 MeV. The red curve shows the total cross section, and the blue and green curves are integrated up to first and second Bragg orders, respectively. In black is an integration over forward-directed angles limited to 1 mrad.

In analyzing the high-energy UED images, it is instructive to first consider the energy dependence of the scattering cross section. This quantity is crucial for predicting diffraction pattern intensity at higher energy. We can express the differential cross section for elastic scattering from an atom as a function of the atomic number *Z* and momentum transfer s=4π sin(θ/2)/λ,

$$\frac{d\sigma }{d\Omega }=\frac{4Z^{2}}{s^{4}a_{0}^{2}}\frac{1-\beta^{2}\;{\text{sin}}^{2}(\theta /2)}{1-\beta^{2}}{\left[1-F{\left(s\right)}^{2}\right]}^{2},$$ (11)

where *a*_0_ is the atomic in Bohr radius and $F(s)=\sum_{i=1}^{3}\frac{A_{i}\alpha_{i}^{2}}{s^{2}+\alpha_{i}^{2}}$ is a function that depends on the details of the screened atomic potential. For gold (Z = 79), $\alpha_{i}=\left[22.864,3.6914,1.4886\right]$ Å^–1^, and $A_{i}=\left[0.2289,0.6114,0.1597\right]$.^3,35,36^ When considering the structure factor of the crystalline lattice, the cross section is heavily weighted by forward-directed rays at the Bragg angles. In Fig. 9, we show the total cross section and compare it with forward-directed cross sections integrated up to integer multiples of λ/a, to represent the intensity of a Bragg peak order. Integration up to a given 1 mrad angle is also shown to exemplify that as the energy increases, the solid angle into which most particles are scattered shrinks. However, the key feature is that cross sections integrated over different annuli, normalized by the momentum factor γβ, are constant, therefore implying that the Bragg order diffracted intensities will have little variation when increasing the beam energy from 3.1 to 8.2 MeV. To verify this, we computed the relative intensity of first-order Bragg peaks in relation to the central beam for both low and high energy by summing pixel counts around the respective peaks on the detector screen. On average, the first-order peaks at high (low) energy exhibit an intensity of 5.2% ± 1.0% (5.6% ± 0.9%) compared to the transmitted beam. These values are well within the experimental uncertainty range, and fully consistent with the expectations.

**FIG. 10.**
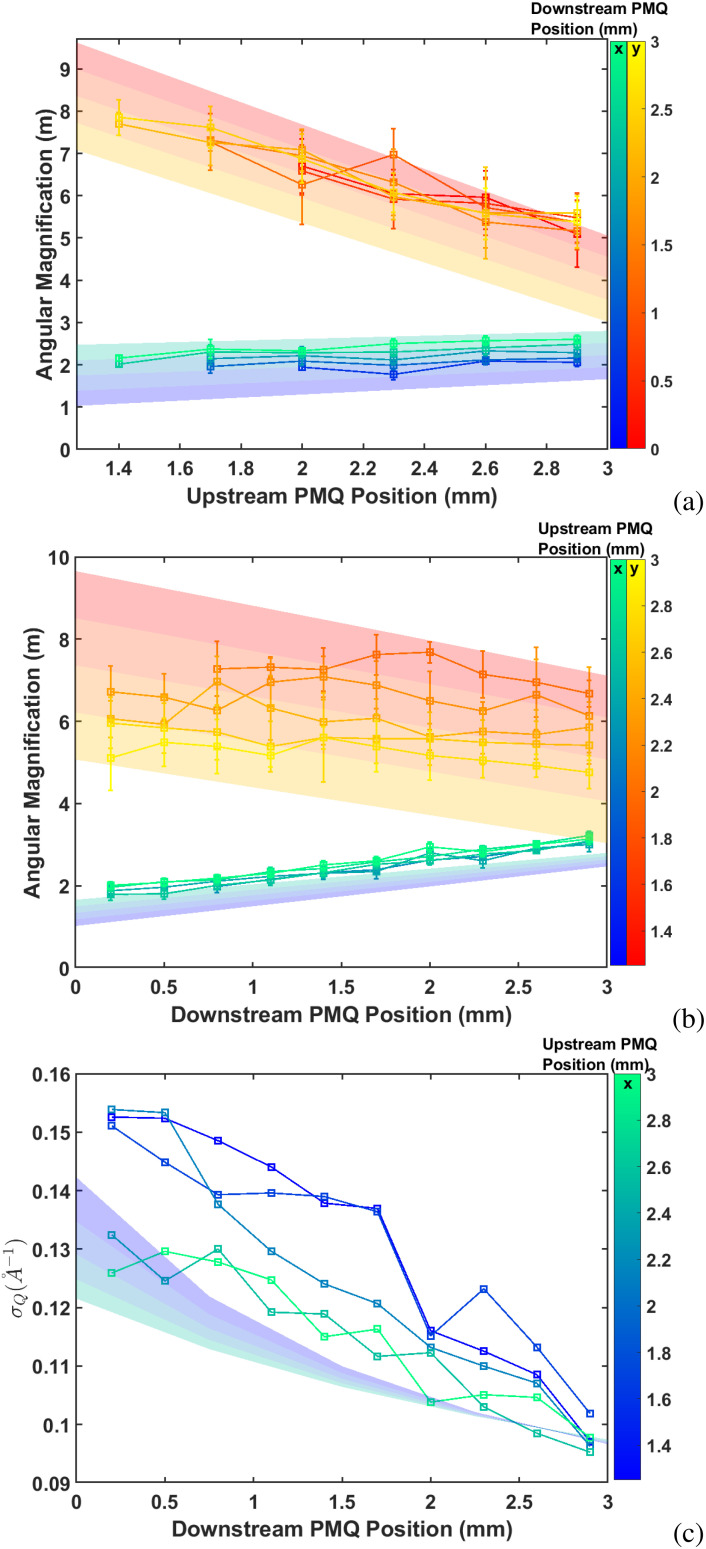
Scan results where spacing of (a) upstream PMQ spacing and (b) downstream PMQ spacing is decreased from 3 mm. (c) Horizontal Q-resolution during the scan.

We then varied the spacings of the PMQ triplet eyepiece over the allowed ranges on the flexure stage to observe how the magnification and the q-resolution would change in the *x* and *y* directions. The upstream PMQ position could be from 1.5 to 3 mm axially, while the downstream PMQ could be translated from 0 to 3 mm. Note that while axially translating the downstream PMQ across its entire range, the centroid of the main beam remained within a region of radius 500 μm on the detector. Given the focal length of the downstream PMQ is approximately 1.8 cm, and the distance to the detector is 17.5 cm, we can estimate the alignment offset as 50 *μ*m in agreement with the expectations from the pulse-wire alignment data. The results corresponding to the PMQ distances scan while maintaining all the other quads fixed are plotted in Figs. 10(a) and 10(b) for the upstream and downstream quad, respectively. The data are color-coded with the position of the other quadrupoles and the results of simulations are overlayed on top of the data points. In agreement with the simulation and linear transport calculations, it is observed that the upstream PMQ position mainly controls the y-angular magnification, while the downstream one has a larger effect on the horizontal one. The most symmetric configuration, yielding an average magnification of 4, is obtained for both quadrupoles at the 3 mm range.

The corresponding q-resolution for each magnification setting can also be estimated from the images by calculating the ratio of the Bragg peak width to their distance from the main beam. We plot in Fig. 10(c) the retrieved horizontal q-resolution along the x-direction as a function of the downstream PMQ position. In agreement with (10), as the magnification gets larger, the q-resolution improves proportionally. The behavior of the data are well captured by a simulation fit where we used the measured spot size at the sample (530 *μ*m) and a fit value of 30 *μ*rad for the rms intrinsic beam angular divergence. A point spread function of 50 *μ*m for the detector screen is also assumed.

Note that the absolute value of q-resolution is affected not only by the beamline optics, but also by factors such as initial beam emittance, energy spread, and point spread function of the screen which were not optimized in these UED experiments. For example, no apertures are positioned upstream of the sample, and the beam emittance was not minimized. For this reason, the data reported is mainly useful to demonstrate the relative resolution improvements enabled by the post-sample magnification optics, rather than an attempt at achieving best absolute resolution. Looking at Eq. (10), even in the case of an optimized optical system, the ultimate q-space resolution is strongly dependent on the beam quality. For example, if the spot size on the sample were to be reduced by a factor of 10, in order to maintain the same q-space resolution, then the emittance of the source would need to be reduced by a factor of 10.

## V. PMQ ABERRATIONS

During the experimental run, distinct distortions were observed in the diffraction pattern as the beam traversed the PMQ aperture using an upstream steering magnet. Images were collected at various steering setpoints, enabling the quantification of dominant lens aberration coefficients and a comparison with the effect on the beam dynamics from the higher order multipole moments present in the Radia model of the PMQs.

The Fourier decomposition of transverse fields in Radia reveals in fact a residual octupole moment, originating from the finite number of magnetized sharp wedges used in the Halbach configuration. Given the normal orientation of the PMQs and associated magnetization symmetry, we anticipated the x-component of the magnetic field to have an expansion in terms of sines while the y-component can be written as a sum of cosine harmonics. The Radia field map validates this expectation when we project the extracted field components onto these harmonics

$$b_{y,n}(r,z)=\frac{1}{\pi }\int_{0}^{2\pi }B_{y}(r,\theta ,z)\;\text{cos}\left(n\theta \right)d\theta .$$ (12)

**FIG. 11.**
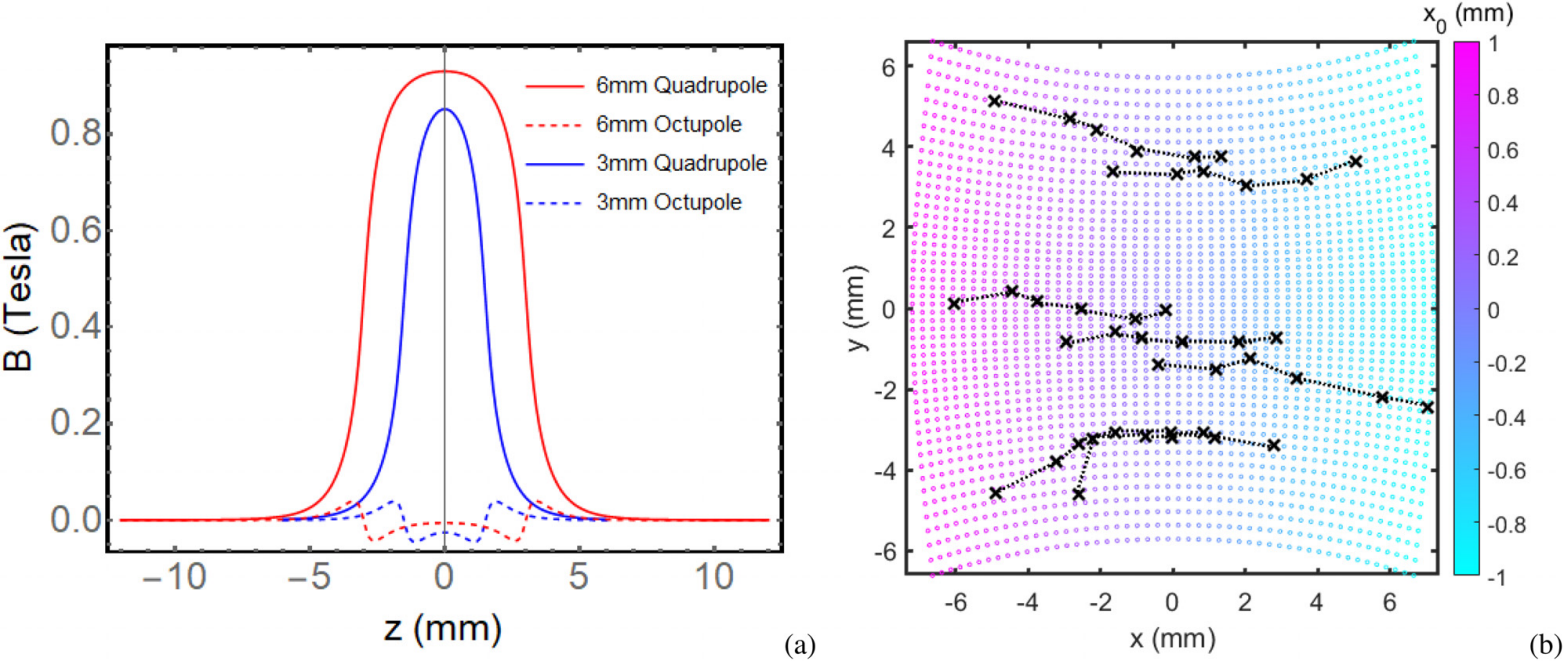
(a) Quadrupole and octupole Fourier components and their axial dependence are shown for the 3 and 6 mm PMQs. (b) Overlay of horizontally steered diffraction beamlet centroid positions, as measured on the detector, with a 3rd-order aberrated image of an initially square grid of rays. The initial positions of the square grid fill the expected region of the first-order Bragg peaks at the objective back focal plane.

Results of the Fourier decomposition at *r* = 1.4 mm at each axial plane are shown in Fig. 11(a). The dominant term as expected is the quadrupole, but a non-zero $b_{x,3}=b_{y,3}=b$ octupole moment is found. The non-linear fields lead to third-order ray equations and a third-order transport map

$$x=C_{x}x_{0}+S_{x}x_{0}^{\prime }+U_{1111}x_{0}^{3}+U_{1133}x_{0}y_{0}^{2}+\cdots ,$$ (13)

$$y=C_{y}y_{0}+S_{y}y_{0}^{\prime }+U_{3333}y_{0}^{3}+U_{3311}x_{0}^{2}y_{0}+\cdots ,$$ (14)

where $U_{1111}=0.25$ mm^−2^, $U_{1133}=2.20$ mm^−2^, $U_{3333}=-11.80$ mm^−2^, and $U_{3311}=-1.18$ mm^−2^ are the third-order transport coefficients that can be evaluated using Green's function approach as detailed in the Appendix. Since the beam is collimated at the objective back focal plane, terms dependent on the angles at the eyepiece location are less prominent. All the ten unique third-order aberration coefficients for both planes are listed in the Appendix, Table II.

In Fig. 11(b), the positions of the Bragg peaks at each steering setpoint, as measured on the detector, are superimposed with the results from the transport, including third-order components. The figure illustrates how a grid of axial rays is distorted, aligning qualitatively and quantitatively with the observed distortions of the Bragg peaks. As these Bragg peaks are steered horizontally across the PMQ entrance, they trace out a path on the detector screen (identified by the dash lines in Fig. 11(b) that follows the pincushion distortion arising from *U*_3311_, confirming the analysis of the PMQ field map. While it is straightforward to take into account these aberrations in post-processing, there are different strategies to minimize them. One option is to optimize the PMQ design by increasing the aperture size reducing the sampling of the higher-order moments in the PMQ magnetic field. This would also mitigate the clipping of the Bragg peaks and maximize the reciprocal space field of view. Alternatively, the defocus of the imaging stage can be optimized to mitigate the impact of the third-order terms on the resolution–an approach commonly used to deal with spherical aberration in TEMs.^37,38^ However, this method alone does not fully compensate the effects of non-linearities in the transport; in order to achieve full aberration correction, higher-order optics would be needed.

## VI. CONCLUSION AND OUTLOOK

In conclusion, we have successfully implemented a compact optical system based on permanent magnet quadrupole (PMQ) optics for ultrafast electron diffraction. The use of strong gradient PMQ focusing enables achieving high angular magnifications within a small footprint. Precise alignment and motion control of the PMQ positions contribute to the flexibility and tunability of the optical system. Drawbacks of the high focusing gradients include the limited beam acceptance due to the small aperture of the optics and the distortions and aberrations resulting from the higher order field components. Still, the strong lenses provide magnification factors as large as 8 and improved q-resolution, which might be critical for some systems to observe dynamics at small scattering vectors (i.e., long spatial scales).

Our approach diverges from traditional methods in the diffraction community, where post-sample optics are rarely employed to observe diffraction patterns. Instead of allowing the beam to just diffract and drift to the detector, our system's capabilities become particularly useful at higher energy levels. This is crucial, significantly where diminishing Bragg angles pose challenges in achieving sufficient separation within a short distance from the beam intrinsic angular spread. The use of optics becomes critical to overcome these challenges and obtain a clear diffraction pattern at 8.2 MeV kinetic energy, nearly twice the energy of other relativistic UED beamlines to date.

Higher beam kinetic energy is expected to enhance diffraction resolution because of shorter electron wavelengths, smaller geometric emittance, and deeper sample penetration, providing wider opportunities to extract information from the sample. Additionally, higher beam energies further suppress space charge effects and allow for more sophisticated phase space manipulations, thus opening the opportunity for shorter beams and smaller spot sizes in future UED instrumentation.

## Data Availability

The data that support the findings of this study are available from the corresponding author upon reasonable request.

## APPENDIX: COMPUTATION OF ABERRATIONS

An approximate magnetic scalar potential (neglecting axial derivatives) that captures the character of the aberrations of the PMQs is

$$V=\mathrm{Gxy}+H(x^{3}y-xy^{3}),$$
(A1)

where, at any particular axial plane, $H\approx b/r^{3}$. We find the axial dependence of *H* is related to the second derivative of *G*.

The equations of motion in that potential are given by

$$x^{\prime \prime }+\kappa x=h(x^{3}-3xy^{2}),$$
(A2)

$$y^{\prime \prime }-\kappa y=h(y^{3}-3x^{2}y).$$
(A3)

h=H/[Bρ] and *H* is the octupole gradient function.

**TABLE II.** sin(4θ) aberration coefficients.

*U*_1111_	$\int_{z_{0}}^{z}g_{x}hC_{x}^{3}\;ds$	*U*_3113_	$-3\int_{z_{0}}^{z}g_{y}hC_{x}^{2}C_{y}\;ds$
*U*_1112_	$3\int_{z_{0}}^{z}g_{x}hC_{x}^{2}S_{x}\;ds$	*U*_3123_	$-6\int_{z_{0}}^{z}g_{y}hC_{x}S_{x}C_{y}\;ds$
*U*_1122_	$3\int_{z_{0}}^{z}g_{x}hC_{x}S_{x}^{2}\;ds$	*U*_3223_	$-3\int_{z_{0}}^{z}g_{y}hS_{x}^{2}C_{y}\;ds$
*U*_1222_	$\int_{z_{0}}^{z}g_{x}hS_{x}^{3}\;ds$	*U*_3333_	$\int_{z_{0}}^{z}g_{y}hC_{y}^{3}\;ds$
*U*_1133_	$-3\int_{z_{0}}^{z}g_{x}hC_{x}C_{y}^{2}\;ds$	*U*_3114_	$-3\int_{z_{0}}^{z}g_{y}hC_{x}^{2}S_{y}\;ds$
*U*_1233_	$-3\int_{z_{0}}^{z}g_{x}hS_{x}C_{y}^{2}\;ds$	*U*_3124_	$-6\int_{z_{0}}^{z}g_{y}hC_{x}S_{x}S_{y}\;ds$
*U*_1134_	$-6\int_{z_{0}}^{z}g_{x}hC_{x}C_{y}S_{y}\;ds$	*U*_3224_	$-3\int_{z_{0}}^{z}g_{y}hS_{x}^{2}S_{y}\;ds$
*U*_1234_	$-6\int_{z_{0}}^{z}g_{x}hS_{x}C_{y}S_{y}\;ds$	*U*_3334_	$3\int_{z_{0}}^{z}g_{y}hC_{y}^{2}S_{y}\;ds$
*U*_1144_	$-3\int_{z_{0}}^{z}g_{x}hC_{x}S_{y}^{2}\;ds$	*U*_3344_	$3\int_{z_{0}}^{z}g_{y}hC_{y}S_{y}^{2}\;ds$
*U*_1244_	$-3\int_{z_{0}}^{z}g_{x}hS_{x}S_{y}^{2}\;ds$	*U*_3444_	$\int_{z_{0}}^{z}g_{y}hS_{y}^{3}\;ds$

We utilize the Green's function method to assess aberrations stemming from the newly introduced terms in the ray equation. The Green's functions are distinctive superpositions of principal rays. For example, the unique ray is expressed as

$$g(z,s)=\{\begin{array}{cc} 0 & \text{if}\;z<s,\\ C(z)S(s)-S(z)C(s) & \text{if}\;z\geq s. \end{array}$$
(A4)

This ray is designed so that at s = z, the ray exhibits no initial offset but has a unit angle [confirmed by the axial derivative of *g* being the Wronskian of the principle rays W(C,S)=1]. Corrections to the linear transport are computed by integrating third-order driving terms in linear order, weighted by the green's function. The corrections corresponding to the octupole are as follows:

$$\delta x=\int_{z_{0}}^{z}\left(x^{3}-3xy^{2}\right)hg_{x}ds,$$
(A5)

$$\delta y=\int_{z_{0}}^{z}\left(y^{3}-3x^{2}y\right)hg_{y}ds.$$
(A6)

In this context, *z*_0_ denotes the objective's back focal plane, while *z* signifies the position of the detector or any point in between. The values of *x* and *y* within the integral are determined linearly using Eqs. (6) and (7). By substituting and expanding the third-order polynomial, we obtain third-order terms that are proportional to combinations of product trios involving *x*_0_, *y*_0_, $x_{0}^{\prime }$, and $y_{0}^{\prime }$. The coefficients of each term are also proportionally related to integrals involving trios of principal rays associated with their respective initial conditions. These coefficients are labeled as *U_ijkl_* where *i* takes on either 1 or 3. The indices *j*, *k*, and *l* range from 1 through 4; for example, 1 and 2 points to *C_x_* and *S_x_*, while 3 and 4 points to *C_y_* and *S_y_*. The superscript corresponds to the plane on which the corrections are applied. Examples of all the relevant aberration coefficients for the sin(4θ) octupole are provided in Table II.
